# Technical note: can resting state functional MRI assist in routine clinical diagnosis?

**DOI:** 10.1259/bjrcr.20180030

**Published:** 2018-06-30

**Authors:** Paula Harman, Christine Law, Shahina Pardhan, ZhiHao Henry Lin, Mark Johnson, Silke Walter, Klaus Fassbender, Richard Aspinall, Iris Q Grunwald

**Affiliations:** 1 Department of Neuroscience, Faculty of Medicine, Anglia Ruskin University, Chelmsford, Essex, , UK; 2 Research and Development, Southend University Hospital NHS Foundation Trust, Essex, UK; 3 Department of Anesthesiology, Perioperative and Pain Medicine, Stanford University, Stanford, CA, USA; 4 Vision and Eye Research Unit (VERU), Faculty of Medicine, Anglia Ruskin University, Cambridge, , UK; 5 Symbolic Systems Program Department, Stanford University, Stanford, CA, USA; 6 Radiology Department, Southend University Hospital NHS Foundation Trust, Essex, UK; 7 Department of Neurology, Saarland University Medical Center, Homburg, Germany

## Abstract

Despite some differences in clinical presentation, it is often difficult to differentiate between dementia with Lewy bodies (DLB), clinical Alzheimer’s dementia (AD) and Parkinson’s disease dementia. However, differentiation can be crucial, especially as patients with DLB characteristically have a hypersensitivity to most antiemetic and neuroleptic drugs as they affect the cholinergic and dopaminergic system, potentially leading to life-threatening catatonia, loss of cognitive function and muscle rigidity. The aim of this study is to evaluate if resting state (RS) functional MRI (fMRI) can be used in routine practice on a 1.5 T scanner to differentiate between AD and DLB on an individual basis. We age- and gender-matched a known DLB patient with an AD patient and a human control (HC). Individual independent component analysis was carried out. Region of interest seeds were chosen from the midcingulate and insula regions. Functional connectivity from insula to midcingulate and within the midcingulate network (part of the Salience network) was lower in DLB than AD or HC. RS-fMRI on a 1.5 T scanner, in a routine clinical setting, detected abnormal functional connectivity patterns and allowed differentiation of DLB and AD in a routine clinical setting. This is the first evaluation of RS-fMRI in a routine clinical setting. It shows that incorporating RS-fMRI into the clinical scanning protocol can assist in early diagnosis and likely assist in monitoring the natural history of the disease or disease modifying treatments.

## Introduction

Despite some differences in the clinical presentation, it is often difficult to differentiate between dementia with Lewy bodies (DLB) and clinical Alzheimer’s dementia (AD) or Parkinson’s disease dementia.^1, 2^

A prominent example is that of the actor Robin Williams. With his first symptoms occurring in October 2013 (sudden spikes in anxiety, constipation, urinary difficulty, heartburn, insomnia, a poor sense of smell, stress, and a slight tremor in his left hand), he eventually suffered from paranoia, delusions, severe insomnia, memory loss, and high cortisol levels indicating stress, before he committed suicide in August 2014.^3^ Originally diagnosed with Parkinson’s disease, his autopsy revealed that he had been suffering from DLB, explaining the underlying cause for his Parkinsonism, depression and memory loss.

In DLB, there is an accumulation of clumps of alphasynuclein (Lewy bodies) in the neurons, first described in 1912 by Friedrich Lewy.^4^ Typical Parkinsonian symptoms in DLB include reduced facial expression, stiffness, shuffling gait with minimal arm swing, dysphonia, dysphagia and hypersalivation, whilst tremors are less common. Early differentiation is important as patients with DLB characteristically have a hypersensitivity to most antiemetic and neuroleptic drugs. These affect the cholinergic and dopaminergic system, potentially leading to life-threatening catatonia, loss of cognitive function and muscle rigidity. Some antidepressants and over-the-counter drug cold remedies may cause acute confusion, delusions, and hallucinations.^2^

A method that has been hailed as a potential biomarker for the early diagnosis of neurodegenerative disease is resting state functional MRI (RS-fMRI),^5^ but it has not yet been implemented into a routine clinical setting. Instead of analysing a cohort of patients, this study intended to investigate the different functional connectivity (FC) patterns between AD, DLB and hehuman controls (HC) on an individual base to assist the early differential diagnosis of dementia.

## Methods and Materials

### Subjects

Subjects with mild severity of symptoms were chosen to best match the cohort that would necessitate differentiation. Selected subjects were recruited as part of a larger observational, cross-sectional study (NCTO2736396 clinicaltrials.gov), reviewed and approved by our local ethics committee, and all subjects gave informed consent for participation.

In order to see if previously reported group findings could be transferred to individual patients in a real-world clinical setting, we selected an age- and gender-matched known DLB patient, AD patient and a healthy control. All subjects were right-handed. The AD patient was on donepezil, and the DLB patient on rivastigmine.

Patients were recruited from the memory clinic or neurology clinic at  Southend University Hospital NHS Foundation Trust. The diagnosis was made on the basis of the clinical symptoms. Blood tests and MRI were performed to rule out other possible causes.

#### Subject 1 (AD)

The AD patient met National Institute of Neurological and Communicative Diseases and Stroke/Alzheimer’s Disease and Related Disorders Association criteria for probable AD.^6^ Symptoms started in his mid-50’s with forgetfulness and general poor short-term memory issues, which were a drastic change from his previous high level of functioning. A diagnosis of probable AD was given at age 60 with worsening short-term memory, some episodes of disorientation and some changes in personality. MRIshowed progressive atrophy around the medial temporal lobes and also the hippocampus.

#### Subject 2 (DLB)

The DLB participant’s symptoms started at the age of 52 with forgetfulness, disorientation, and fluctuating cognition with great variations in attention, which sometimes left him being unable to respond to those around him. Probable DLB was diagnosed at age 63 after symptoms included visual hallucinations, visuoperceptive difficulties and fluctuating cognition, the patient now meeting the consensus criteria for probable DLB which include the presence of two or more of the core clinical features: fluctuating cognition, visual hallucinations and/or Parkinsonism.^2^

#### Subject 3 (HC)

The control was a right-handed, white British male with 22 years of education (school, college, university and post-graduate education). The participant’s known medical history consisted of coeliac disease, erythromelalgia and borderline rheumatoid arthritis.

### Neuropsychological evaluations

The Mini Mental State Examination (MMSE) was used to assess global cognition, whilst episodic memory was assessed with the Rey Auditory Verbal Learning Test (RAVLT). The Stroop test was used as a test of executive function, attention and concentration, with block colour, congruent colour–word pair, and incongruent colour–word pair tasks being completed. Finally, visual short-term memory (VSTM)  was assessed using the VSTM assessment created by the Vision and Eye Research Unit at Anglia Ruskin University. Task 1 assessed object memory, Task 2 assessed location memory, and Task 3 assessed object-location memory.

Additionally, the clinical dementia rating (CDR) was completed as a semi-structured interview to assess the severity of dementia and the Beck Depression Inventory—Second Edition (BDI-II) was used to identify depression.

### Image acquisition

All structural and functional images were acquired on non-sedated patients on the same clinical 1.5 T MRI scanner (GE Signa Excite HDXT), not optimized for research, using an 8-channel high-resolution parallel imaging head coil. Subjects were instructed to lie still in the scanner and not to think of anything whilst keeping their eyes closed.

High-resolution *T*_1_ weighted three-dimensional fast spoiled gradient echo images were taken in order to obtain anatomical images for core registration of the fMRI data, covering the head from foramen magnum to vertex. Voxel size of fast spoiled gradient echo images was 1 × 1 × 1 mm^3^.

An echo-planar imaginggradient echo sequence was used for the RS functional sequence. The number of volumes (time frames) collected was 200 with a field of view of 24 cm and a matrix size of 64 × 64. Number of slices was 32, slice thickness 5 mm (gap between slices = 0.5 mm), repetition time = 2.5 s. Average acquisition time was 13 min.

### Functional imaging analysis

No sequences needed repeating. RS-fMRI data were first corrected from cardiac and respiratory physiological noise using RETROICOR^7^ and RVHRCOR.^8^ Data preliminary processing was carried out using FMRIB Software Library (FSL):^9^ (1) slice timing correction for interleaved acquisitions; (2) head motion correction via a robust and accurate intramodal volume linear registration (MCFLIRT); (3) spatial smoothing with a 5 mm FWHM Gaussian kernel; (4) high-pass temporal filtering (Gaussian-weighted least-squares straight line fitting, with σ = 100 s); (5) spatial normalization via estimating a B-spline basis transformation from an individual functional space to MNI152 standard brain space (FLIRT).

Individual independent component analysis (ICA) was carried out using FSL Multivariate Exploratory Linear Decomposition into Independent Components. Each patient’s independent components were carefully inspected and noise components were noted and further by FSL function fsl_regilt to regress out noise components using ordinary least squares.

Region of interest (ROI) seeds were chosen from the midcingulate and insula regions, which are part of the Anterior Salience Network. ROI masks were obtained from findlab.stanford.edu.^10^ Mean time series from each ROI was used as input regressor to FSL FEAT in order to identify other voxels having similar spontaneous time course. Resulting z-statistic was threshold at 3.5.

## Results

### Neuropsychological evaluations

Regarding global cognition, the DLB patient scored the lowest with an MMSE score of 20, the AD patient scoring 25. The DLB patient had the highest scores on CDR global (1) and sum of boxes (5) assessment, showing a mild severity stage of dementia. The AD patient had a CDR global score of 0.5, landing him in the early disease stage of “questionable” severity.

Whilst the episodic memory, as measured by the encoding, immediate and delayed recall sections of the Rey Auditory Verbal Learning Test, was low in the DLB patient (encoding 25, immediate recall 4, delayed recall 2, recognition 4), the AD patient had the lowest scores for: immediate (0) and delayed recall (1) and scored poorly in recognition (4) and encoding (16).

The DLB patient, when compared to the AD patient and HC, had the worst performance on the Stroop tasks: block (155 * vs * 49 * vs * 34 s), congruent (159 * vs * 84 * vs * 22 s) incongruent (303* vs *81 * vs * 54 s), whilst also making the only errors in the block colour (1) and incongruent colour-word tasks (11).

The DLB patient was unable to complete any of the VSTM tasks, as he did not meet the visual acuity requirements. The AD patient scored low on all VSTM tasks (Task 1: 81.25%, Task 2: 75.00%, Task 3: 50.00%), the HC reaching normal scores (Task 1: 93.75%, Task 2: 87.50%, Task 3: 62.50%).

BDI-II score in the DLB patient was 18, suggesting mild (BDI-II 14–19) depressive symptoms and 4 in the AD patient suggested minimal (BDI-II 0–13) depressive symptoms. The HC scored 0 on the CDR global and CDR sum of boxes and BDI-II assessments.

Neuropsychological findings are listed in Table 1.

**Table 1:** Demographic data and neuropsychological scores

		**AD**	**DLB**	**HC**
Age		63	63	64
Education (years)		24	12	22
Global cognition	MMSE	25	20	28
Severity of dementia	CDR global score	0.5	1	0
CDR sum of boxes	4	5	0
Episodic memory	RAVLT—encoding	16	25	48
RAVLT—immediate recall	0	4	9
RAVLT—delayed recall	1	2	8
RAVLT—recognition	4	4	12
Executive function, attention and concentration	Stroop—block (seconds)	49	155	34
Stroop—block (errors)	0	1	0
Stroop—congruent (seconds)	84	159	22
Stroop—congruent (errors)	0	0	0
Stroop—incongruent (seconds)	81	303	54
Stroop—incongruent (errors)	0	11	0
Visual short-term memory	VSTM Task 1 (percentage)	81.25	–^*a*^	93.75
	VSTM Task 2 (percentage)	75	–	87.50
	VSTM Task 3 (percentage)	50	–	62.50
Depression	BDI-II	4	18	0

AD, Alzheimer disease; BDI-II, Beck Depression Inventory; CDR,clinical dementia rating; DLB, dementia with Lewy bodies; MMSE, Mini-Mental State Examination; RAVLT, Rey Auditory Verbal Learning Test; VSTM, Visual Short-Term Memory.

BDI-II, 0–13: minimal depression; 14–19: mild depression; 20–28: moderate depression; 29–63: severe depression.

CDR: 0 = normal; 0.5 = very mild dementia; 1 = mild dementia; 2 = moderate dementia; 3 = severe dementia.

^*a*^ The DLB patient was unable to complete the VSTM tasks as did not meet the visual acuity requirements.

### RS-fMRI results

#### Salience network

**Figure 1.**
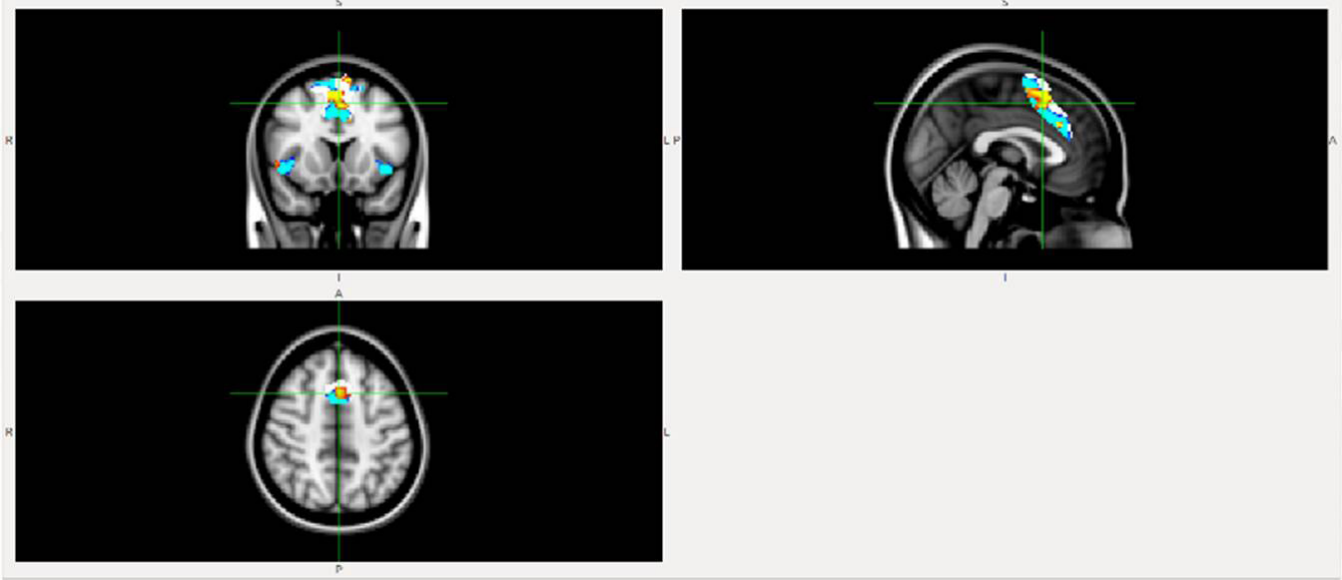
FC within the midcingulate network (part of Salience network) is lower in DLB than AD. White, midcingulate network mask; blue, AD; red-yellow, DLB. AD, Alzheimer disease; DLB, dementia with Lewy bodies; FC, functional connectivity.

**Figure 2.**
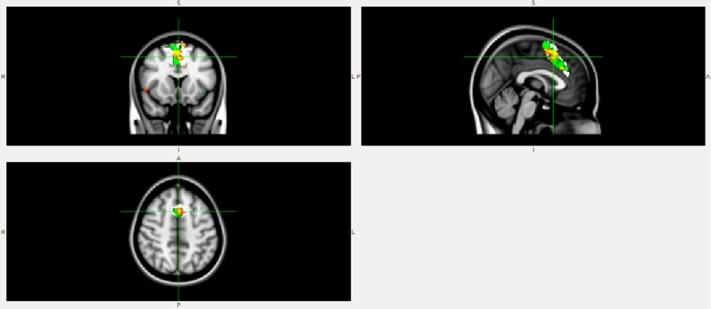
FC within the midcingulate network (part of Salience network) is lower in DLB than HC. White, midcingulate network mask; Blue, HC; red-yellow, DLB. DLB, dementia with Lewy bodies; FC, functional connectivity.

**Figure 3.**
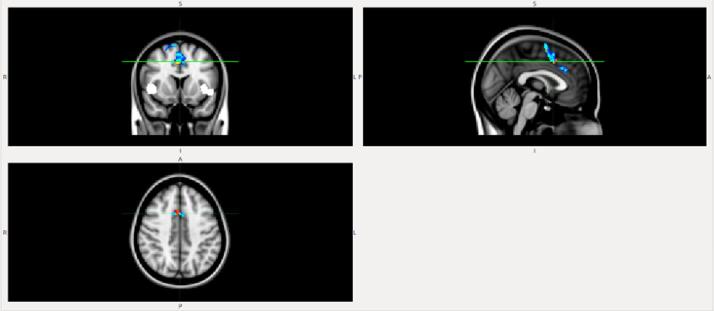
FC from insula to midcingulate is lower in the patient with DLB than in the patient with AD. White, insula mask; Blue, AD; red-yellow, DLB. AD, Alzheimer disease; DLB, dementia with Lewy bodies; FC, functional connectivity.

**Figure 4.**
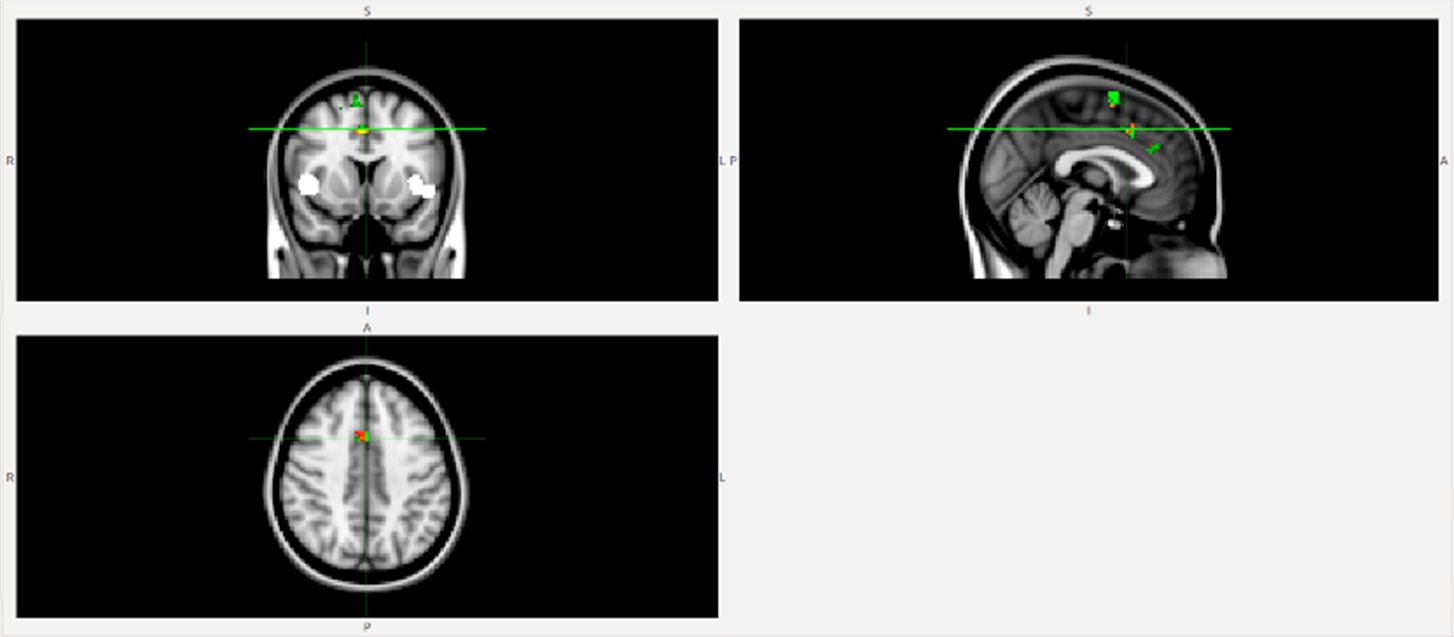
FC from insula to midcingulate is lower in the patient with DLB than in the HC. White, insula mask; green, HC; red-yellow, DLB, dementia with Lewy bodies.

Individual ICA from the ROI seeds from the midcingulate and insula regions showed:

1. FC within the midingulate network (part of anterior Salience network) was lower in the DLB patient compared to the AD patient (Figure 1) and HC (Figure 2).
2. FC from insula to midingulate was lower in the DLB patient compared to the AD patient (Figure 3) and HC (Figure 4).

## Discussion

As the overlap of neuropathological, cognitive, emotional and motor symptoms make an accurate differential diagnosis difficult, DLB is often confused with Parkinson’s disease dementia, AD and/or vascular dementia, especially in the early stages of the disease. However, differentiation is important due to the characteristic sensitivity of DLB patients to most neuroleptic and antiemetic drugs.^1^

Up to now, the neural changes responsible for the symptoms of attention deficits, motor features of Parkinsonism and depression that are characteristic of DLB are not well-understood.^11^ Characteristic clinical symptoms associated with DLB are believed to correlate with FC within specific RS networks.^11^ For example, Parkinson like symptoms in DLB correlate with structural pathology and neurotransmitter abnormalities in the Putamen,^12^ whilst emotional regulation and the high incidence of depression seen in DLB is associated with Basal ganglia and limbic networks, specifically the caudate.^13^ Fluctuating cognition, another frequent symptom of DLB,^2^ is linked to the key role the thalamus plays in maintaining consciousness.^14^ Attention deficits are greater in DLB than AD patients and are linked to the Salience and Executive networks.^15^ Depending on the severity and symptom profile, the salience, executive control, default mode network (DMN), basal ganglia and limbic networks should thus be more affected in DLB patients than in AD or HCs.

There are only a few studies^16–19^ that have investigated FC in DLB and none have looked at its value to assist in the differential diagnosis on an individual patient level. All previous analyses were done at a group level. In our analysis we tested the discriminative utility of RS-fMRI at the individual patient level, attempting to translate previous research findings into clinical practice with RS-fMRI as a potential further diagnostic biomarker.

Franciotti et al^17^ previously utilized the ICA method in DLB patients, but only described a lack of abnormalities in the DMN connectivity. No analysis of the salience network was reported. Galvin et al^16^ showed both increased FC with putamen and parietal regions and decreased connectivity with prefrontal and primary visual cortices when focusing solely on precuneus connectivity and using the whole structure as the seed region. Lowther et al^11^ showed less FC in DLB compared with AD patients and HC in the posterior DMN and increased FC in the posterior cingulate and putamen.

In our study, we followed a seed-based approach, with special focus on the salience network adapted from Lowther et al.^11^ ROI seeds were chosen from the midcingulate and insula regions (part of the Anterior Salience Network). Our study showed that the DLB patient distinctly differed in FC within the salience network compared to the AD patient and control, with decreased FC from insula to midcingulate and within the midcingulate region. Similar to our findings (less connectivity in DLB), previous studies^20^ have shown enhanced FC of salience networks in AD. Lowther et al^11^ has previously described less FC in a DLB group compared to AD and control groups in a number of brain regions. The authors had identified three ICs (IC 3,5,18) corresponding to brain regions known to be involved in salience and executive control.

The lower FC in the salience network in the DLB patient compared with HC and AD could be related to the characteristic symptoms we observed. Clinically, when compared to the AD patient, the DLB patient had severe impairment in executive function, attention and concentration, which was mirrored in a very poor performance on the Stroop tasks, both regarding time and errors, whilst short-term memory and orientation to time and place remained intact. Our DLB patient had problems with figure copying (visuospatial impairment) and difficulty with serial sevens, most likely as a result of impaired attention. A correlation between severity of symptoms and FC in DLB has previously been described with higher FC in the less affected group.^11^ Interestingly, a recent study in ADHD patients also showed an association with decreased FC between the salience and executive control networks as well as with peripheral brain regions, underpinning the association between attention and the Salience network.^21^

We deliberately chose subjects with mild severity of symptoms, as this would best replicate the cohort of patient that clinically necessitates differentiation. Regarding global cognition, both were not severely affected (MMSE 20 and 25), the DLB patient showing a mild severity stage of dementia, the AD patient being in the early disease stage of “questionable” severity.

Our findings are consistent with previous results in that we also showed less connectivity in DLB subjects compared with controls and AD in the salience network. We were able to identify distinct patterns of activity in FC that may assist in discrimination of dementia with Lewy bodies from Alzheimer’s disease and cognitively normal patients.

### Study limitations

Until autopsy, a definite diagnosis will not be possible. However, experienced clinicians diligently examined all subjects, coming to a consensus diagnosis. No CSF sampling or genetic analysis was performed. However, currently there are no broadly applicable biomarkers for DLB and it seems premature to perform genetic testing in routine clinical setting for the diagnosis of the disease.

Although subjects were well matched for gender, age and handedness and there was no significant impairment in global cognition, the DLB patient had less years of education. Our results show that it was possible to differentiate between DLB, AD and human controls on an individual basis. However, further large scale studies on individually matched patients would be necessary to further validate this method.

Lorenzi et al^22^ previously reported that AD patients on memantine exhibited increased connectivity. In our study, none of the patient were on memantine but they were not medication free. Thus, potential interaction of the medication with FC connectivity cannot be excluded.

The range of the neuropsychological test battery was limited to not significantly exceed 1 h in order to minimize stress and cognitive burden to the patients. They do, however, cover a broad range of cognitive domains and were all conducted by the same experienced rater.

Only 200 acquisitions were obtained to not impact routine clinical scanning and limit the time a patient would need to be in the scanner.

Although subjects were instructed to not think of anything whilst in the scanner, it is impossible to control brain activity. However, we see the advantages of RS-fMRI in its non-invasive nature, the short time of image acquisition (10–13 min), the fact that no specific task is necessary and that patients who are too severely affected to actively cooperate in task-based fMRI study can be successfully evaluated.

We did not perform a correction for atrophy as, in accordance with Balthazar et al,^23^ this would not be useful in the context of finding a biomarker in routine clinical setting.

We only evaluated the salience network, whilst other RS networks might also be affected in DLB and AD. However, the aim of this study was to assess the individual patient, focusing on the patient’s specific symptoms, with the aim of generating data to assist the individual diagnosis. Future cohort studies can focus on the different networks and their role in neurodegenerative disease as well as structural change. A larger data pool from routine clinical practice can further our understanding of neurodegenerative diseases. Possibly, in the future, a common platform collection of biomarkers and outcome measures can be created, which would likely expedite clinical–translational research. Consideration might be given to establishing a national server and creating a plan for reimbursement of biomarkers as well as support for an interdisciplinary collaboration.

## Conclusion

We were able to show that individual measures within the salience network allow differentiation between DLB, AD and normal ageing, even in clinically mild cases, in a routine clinical setting on a 1.5 T scanner. RS-fMRI has the potential to serve as an early biomarker to distinguish AD from DLB and HC. We hope that our work inspires future studies in a routine clinical setting to provide more evidence for the strength of this method.

## Learning Points

1. RS-fMRI can be performed in routine clinical setting on a 1.5 T MRI scanner.
2. RS-fMRI can be useful in differentiating between different neurodegenerative diseases
3. RS-fMRI has the potential to serve as an early biomarker for AD.
